# Iron as diagnostic marker of cancer

**DOI:** 10.1186/1897-4287-13-S2-A5

**Published:** 2015-11-26

**Authors:** Grzegorz Sukiennicki, Magdalena Muszyńska, Katarzyna Jaworska-Bieniek, Katarzyna Kaczmarek, Wojciech Marciniak, Marcin Lener, Katarzyna Durda, Tomasz Gromowski, Tomasz Huzarski, Tomasz Byrski, Jacek Gronwald, Oleg Oszurek, Cezary Cybulski, Tadeusz Dębniak, Antoni Morawski, Anna Jakubowska, Jan Lubiński

**Affiliations:** 1 Department of Genetics and Pathology, International Hereditary Cancer Center, Pomeranian Medical University, Szczecin, Poland; 2 Read - Gene, S.A., Grzepnica, Poland

## 

Iron plays an important role in many metabolic processes, is included in the delivery of oxygen to cells and redox processes. Deficiency or excess of iron can lead to multiple organ failures and in extreme cases to death. The latest studies show that the iron can significantly influence the risk of cancer development and progression. Scientists reported association between high levels of iron in the serum and the risk of colon, liver, stomach and breast cancers. The low iron level was detected in patients with bladder and lung cancers. There are also studies in which authors showed lower iron level in patients with colorectal cancer and breast or they did not find any correlation between iron levels and the risk of breast cancer.

## Aim

The aim of the study is identification of correlations between the serum concentrations of iron and occurrence of laryngeal and colorectal cancers.

## Materials and methods

The subjects of the study were unselected cancer cases from Polish population. Retrospective study - serum was collected during laryngeal cancer or colorectal cancer diagnosis before treatment. One case and one control were matched for year of birth (∓ 3 years), number and location of cancer among Iº relatives, smoking-the number of pack years (∓ 10%), adnexectomy status, CHEK2 mutation to achieve the maximum of similarity between them.

- 109 laryngeal cancer patients paired with the control group 1:1

- 100 colon cancer patients paired with the control group 1:1

The iron was quantitatively measured by ICP-MS (Inductively Coupled Plasma Mass Spectrometry), (model Elan DRC-e 6100 th, PerkinElmer).

The proportion of cases and control in the first quartile was taken as a reference to calculate the odds ratio, confidence interval and p-value of the multivariate conditional logistic regression.

## Results

This study shows that concentration levels of iron in blood serum may be a factor associated with risk and attendance of laryngeal cancer and unselected colorectal cancer patients.

In retrospective group found a 3-fold decrease in the risk of colorectal cancer, the level of iron> 1100 to 2000 ug/l.

## Conclusions

Serum iron level may be a diagnostic factor for cancer in Poland. Low level of iron may be a diagnostic marker for selection of people to colonoscopy.

## Acknowledgements

This work was financially supported by National Science Centre grant no. 2013/11/N/NZ4/02250.

